# In Situ Synthesis of Ag@Cu_2_O-rGO Architecture for Strong Light-Matter Interactions

**DOI:** 10.3390/nano8060444

**Published:** 2018-06-17

**Authors:** Shuang Guo, Yaxin Wang, Fan Zhang, Renxian Gao, Maomao Liu, Lirong Dong, Yang Liu, Yongjun Zhang, Lei Chen

**Affiliations:** 1 College of Physics, Jilin Normal University, Siping 136000, China; guoshuang0420@163.com (S.G.); zhangfan147258@126.com (F.Z.); ren7771993@163.com (R.G.); 18844592029@163.com (M.L.); dlr5640@163.com (L.D.); 2 Key Laboratory of Functional Materials Physics and Chemistry, Ministry of Education, Jilin Normal University, Changchun 130103, China; liuyang@jlnu.edu.cn; 3 College of Chemistry, Jilin Normal University, Siping 136000, China

**Keywords:** nanocomposite, rGO-substrate, Ag@Cu_2_O, interfacial effect

## Abstract

Emerging opportunities based on two-dimensional (2D) layered structures can utilize a variety of complex geometric architectures. Herein, we report the synthesis and properties of a 2D+0D unique ternary platform-core-shell nanostructure, termed Ag@Cu_2_O-rGO, where the reduced graphene oxide (rGO) 2D acting as a platform is uniformly decorated by Ag@Cu_2_O core-shell nanoparticles. Cu_2_O nanoparticles occupy the defect positions on the surface of the rGO platform and restore the conjugation of the rGO structure, which contributes to the significant decrease of the *I*_D_/*I*_G_ intensity ratio. The rGO platform can not only bridge the isolated nanoparticles together but also can quickly transfer the free electrons arising from the Ag core to the Cu_2_O shell to improve the utilization efficiency of photogenerated electrons, as is verified by high efficient photocatalytic activity of Methyl Orange (MO). The multi-interface coupling of the Ag@Cu_2_O-rGO platform-core-shell nanostructure leads to the decrease of the bandgap with an increase of the Cu_2_O shell thickness, which broadens the absorption range of the visible light spectrum.

## 1. Introduction

Since Geim and Novoselov discovered the two-dimensional (2D) electron motion in graphene, 2D materials have attracted attention due to their unique properties and their wide applications in photocatalysis, sensing, detection, and energy storage [1,2,3,4,5,6,7,8]. In recent years, researchers have conducted in-depth explorations of 2D materials to create a large 2D family of materials that mainly include carbon nanomaterials, metal oxides, transition metal dichalcogenides, hexagonal boron nitride, and so on [9,10,11,12,13]. Two-dimensional graphene composing of carbon atoms has attracted high attention due to its excellent physical properties, such as ballistic transport, high Young’s Modulus, and super-high electron mobility. However, the zero-band system properties of graphene prevented it from being directly used in optoelectronic devices and photocatalytic processes which were based on the utilization of carriers. To overcome the limitation of graphene, synergistic effects were recommended by using carboxylic acid groups [14], semiconductors, or noble metal composites [15]. To date, a series of graphene-based composites have been realized, for example, a semiconductor and graphene oxide composite (TiO_2_/GO) [16], noble metal and graphene oxide composite (Ag/GO) [17], hydroxide and graphene oxide composite (Zn(OH)_2_/GO) [18], and multiple structure composite (Ag-ZnFe_2_O_4_@rGO) [19]. These graphene-based composites have excellent electron transport channels and can quickly transfer excitons to suppress the recombination of carriers compared to other carbon nanomaterials [20,21]. Although the synergistic effects bring some advantages, they also undermine the low-dimensional structure of the graphene and it is still difficult to modify the nanostructure system on the surface of 2D graphene, especially the core-shell heterostructure arranged on the surface of graphene. Therefore, it is necessary to create a new composite structure which can not only keep the graphene low-dimensional structure but also connect the isolated nanostructure units together.

Guo et al. reported the ternary Ag-Cu_2_O/rGO with excellent peroxidase-like nanocatalysts, which was based on the Cu_2_O sphere modified by Ag nanoparticles and attached to rGO [22]. However, the surface of Ag nanoparticles are not stable. To overcome the instability of surface Ag nanoparticles, we designed a heterostructure of Ag@Cu_2_O-reduced graphene oxide with the core-shell complex-structure. Ag@Cu_2_O core-shell nanoparticles are considered excellent photocatalytic candidate material, which have excellent performance in photocatalytic degradation of pollutants [23]. The main reasons to use Cu_2_O as a shell structure are: (1) Cu_2_O has a narrow bandgap (2.2 eV) compared with a wide bandgap semiconductor (like ZnO) [24], in which the absorption band is located in the visible light range; (2) Cu_2_O has advantages of excellent catalytic efficiency for C–C, C–N, and C–O bonds. Ag can provide plenty of free electrons and the large energy level difference between the Ag nanoparticles and Cu_2_O facilitates the transfer of photogenerated electrons between the Cu_2_O shell and the Ag core [25,26]. However, the increase of carrier efficiency in a pure core-shell structure is limited, and composite materials with higher efficiencies are still required. Therefore, combining the Ag@Cu_2_O core-shell structure with graphene, which is a superior electron transport material, can both improve the utilization of sunlight and achieve higher carrier transfer efficiency.

In this paper, this was the first time to report the orderly arrangement of Ag@Cu_2_O core-shell structures on an rGO substrate. The rGO nanosheets act as a sheet platform and the Ag@Cu_2_O core-shell nanoparticles are decorating the surface of the rGO, which forms a 2D+0D heterogeneous structure. The broad spectral absorption and enhanced charge transfer efficiency in the Ag@Cu_2_O-rGO platform-shell-nucleus configuration have been observed. We demonstrated that the structure has strong light–matter interactions using photocatalytic degradation of the Methyl Orange (MO). Our study indicates that the Ag@Cu_2_O-rGO platform-shell-core configuration with wide spectral absorption and strong light–matter interactions is a new candidate to be applied in photoanodes, photoelectrocatalysis, and so on.

## 2. Experimental Section

### 2.1. Materials

Potassium persulfate (K_2_S_2_O_8_, AR, Shanghai China), phosphorus pentoxide (P_2_O_5_, AR, Shanghai China), graphite powder, concentrated sulfuric acid (H_2_SO_4_, 98.98 wt %, Shanghai China), potassium permanganate (KMnO_4_, Shanghai China), phosphoric acid (H_3_PO_4_, 85 wt %, Shanghai China), hydrogen peroxide (H_2_O_2_, 30 wt %, Shanghai China), silver nitrate (AgNO_3_, AR, Shanghai China), sodium citrate dihydrate (C_6_H_5_-Na_3_O_7_·2H_2_O, AR, Shanghai China), copper(II) nitrate trihydrate (Cu(NO_3_)_2_·3H_2_O, AR, Shanghai China), polyvinylpyrrolidone (C_6_H_9_NO) n, AR, Shanghai China) and we named as PVP, hydrazine hydrate (H_4_N_2_·H_2_O, 85 wt %, Shanghai China), and anhydrous ethanol (C_2_H_6_O, ≥ 99.8 wt %, Beijing, China) were purchased from Sinopharm Chemical Reagent Co., Ltd. All chemicals were used as received without further treatment. Deionized water was used in all solution preparations in this study.

### 2.2. Preparation of the Ag@Cu_2_O-rGO Composites 

#### 2.2.1. Fabrication and Modification of a 2D Graphene Oxide (GO) Nanosheet

GO nanosheets were synthesized by a two-step oxidation of graphite powder summarized from Hummer’s and Offeman’s method [27]. Then, the surface of the GO sheet was modified with Ag nanoparticles (NPs). First, 1 mg of GO was dispersed in 30 mL of deionized water, and the solution was sonicated for 1 h to obtain homogeneously dispersed GO. The GO solution and 170 mL of deionized water were poured into a three-necked flask, and the solution was uniformly dispersed by magnetic stirring. Then, 36 mg of AgNO_3_ was added to the three-necked flask and slightly boiled at 90 °C for 20 min. Finally, 4 mL of a sodium citrate dihydrate (C_6_H_5_-Na_3_O_7_·_2_H_2_O, 1 wt %) solution was added quickly to the flask, and the solution was incubated at 85 °C for 30 min until the solution became dark green. The Ag-rGO composite structure based on graphene oxide was obtained.

#### 2.2.2. Synthesis of the Ag@Cu_2_O-rGO Composites

To get the Ag@Cu_2_O-rGO structure, our previous synthesis method of Ag@Cu_2_O core-shell structure was improved [28]. The specific method is as follows: 50 mL of 8 mM Cu(NO_3_)_2_ solution was poured into a beaker. Then, 1 g PVP was added to the Cu(NO_3_)_2_ solution, and the solution was magnetically stirred for 5 min to completely dissolve the PVP. Twenty milliliters of the as-prepared Ag-GO solution were centrifuged, washed, and dispersed in the Cu(NO_3_)_2_ solution. Fifty-five microliters of H_4_N_2_·H_2_O (35 wt %) were added to the beaker, and the solution was continuously magnetically stirred (300 rpm) and maintained for 90 s at room temperature. Then, the Ag@Cu_2_O-rGO composite structures formed. Finally, the composite structures were centrifuged, washed, and stored in centrifuge tubes (4 °C). Change the concentration of Cu(NO_3_)_2_ (0 mM, 2 mM, 4 mM, 6 mM, 8 mM, and 10 mM) and the added volume of H_4_N_2_·H_2_O (0, 14, 27, 41, 55, and 68 μL) in the above experiment to acquire a group of Ag@Cu_2_O-rGO composite structures containing different Cu_2_O ratios.

### 2.3. Degradation MO Experiments

The photocatalytic activity of the materials was tested under visible-light irradiation at room temperature using MO as the probe molecule. The detailed steps are as follows. First, 1 mg of the sample was uniformly dispersed in 50 mL of an MO solution (4 mg/L). Then, the material was allowed to adsorb the dye under dark conditions for 40 min to reach adsorption equilibrium before visible-light irradiation. Under constant visible-light irradiation, 2 mL of the mixed solution was removed from the above solution at regular intervals, and the 2 mL sample was centrifuged at 10,000 rpm for 3 min. The absorbance of the resulting supernatant was tested using a Hitachi U-4100 spectrophotometer (Hitachi, Tokyo, Japan). Finally, the initial and final concentrations of the solution were used to calculate the degradation efficiency of MO by the following equation:

*D*% = (*c*_0_ − *c*_t_)/*c*_0_ × 100%

where *c*_0_ is the MO initial concentration, and *c*_t_ is the temporal concentration of MO.

### 2.4. Characterization

Scanning electron microscopy (SEM, JEOL Ltd., Tokyo, Japan) images were obtained by using a JEOL 7800F scanning electron microscope operated at a 5.0 kV accelerating voltage to study the surface morphologies of the samples. Transmission electron microscopy (TEM, JEOL Ltd., Tokyo, Japan) images were obtained by using a Hitachi H-800 transmission electron microscope operated at a 200-kV accelerating voltage to study the crystallization of the nanomaterials. X-ray photoelectron spectroscopy (XPS, Thermo Fisher Scientific, Waltham, MA, USA) was performed by using Thermo Fisher Scientific system to study the elemental composition and chemical state of the samples. UV-Vis (Tokyo, Japan) absorption spectra were obtained by using a Shimadzu 3600 spectrometer to study the optical properties of the structures. The absorption spectra were obtained by a Hitachi U-4100 spectrophotometer to study the concentration of the signal molecule.

## 3. Results and Discussion

### 3.1. Structure and Properties of the Ag@Cu_2_O-rGO Nanocomposites

**Figure 1:**
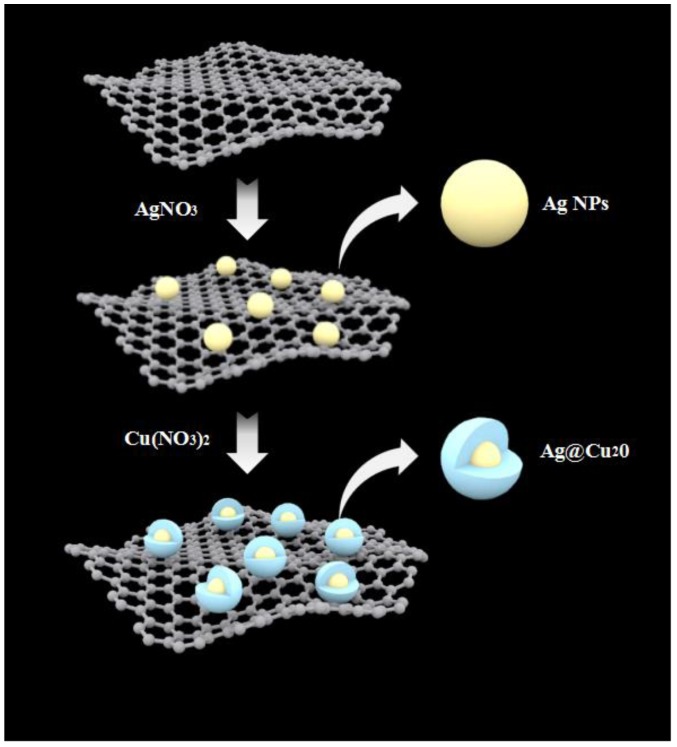
Schematic of the synthetic procedure for Ag@Cu_2_O-reduced graphene oxide (rGO) nanostructures.

**Figure 2:**
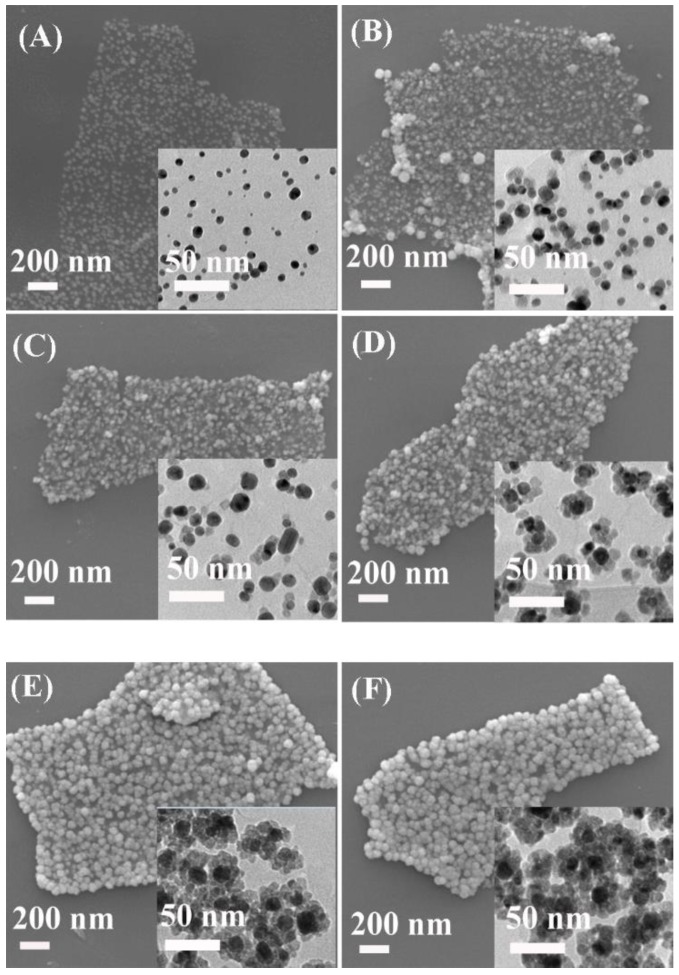
SEM images of (**A**) Ag-rGO and Ag@Cu_2_O-rGO nanocomposites with different concentrations of Cu(NO_3_)_2_ solution ((**B**–**F**), S-2 mM, S-4 mM, S-6 mM, S-8 mM, and S-10 mM). The insets are the corresponding TEM images.

Figure 1 shows the fabrication process for the Ag@Cu_2_O-rGO platform-shell-core structure. First, GO nanosheets acting as the platform for Ag NPs were synthesized by a two-step oxidation of graphite powder. Then, Ag NPs were decorated on the GO nanosheets based on the sol-gel method. Finally, Cu_2_O was reduced in situ on the Ag NP’s surface. The detailed fabrication process is given in Section 2.2 and Figure S1. Figure 2A–F shows the SEM images of the platform-shell-core Ag@Cu_2_O-rGO prepared by reducting Cu(NO_3_)_2_ with different concentrations: 0 mM, 2 mM, 4 mM, 6 mM, 8 mM, and 10 mM, and the samples are labeled as S-0 mM, S-2 mM, S-4 mM, S-6 mM, S-8 mM and S-10 mM, respectively. It can be seen from Figure 2 that all of the samples were decorated by the Ag@Cu_2_O nanoparticles on the micron scale. In addition, the nanoparticles distribute dispersedly on the sheet without obvious agglomeration. Compared to the SEM image of the single GO (Figure S2), the composite Ag@Cu_2_O-rGO show a 2D structure, which indicates that rGO acts as the stable platform for the modification of Ag@Cu_2_O. The sizes of the Ag@Cu_2_O nanoparticles on platform rGO gradually increase as the concentrations of Cu(NO_3_)_2_ increase from 0 mM to 10 mM, as shown in the SEM images. The inset TEM images also show some obvious small Cu_2_O nanoparticles on the surface of Ag when the Cu(NO_3_)_2_ is added, and the perfect shell-core structure of the nanoparticles is found when the concentration of Cu(NO_3_)_2_ is higher than 6 mM, and the thickness of the shell increases from 10 ± 2 nm for S-6 mM to 13 ± 2 nm for S-10 mM, respectively, but the size of core remains unchanged at about 14 ± 3 nm as shown in the Figure S3.

**Figure 3:**
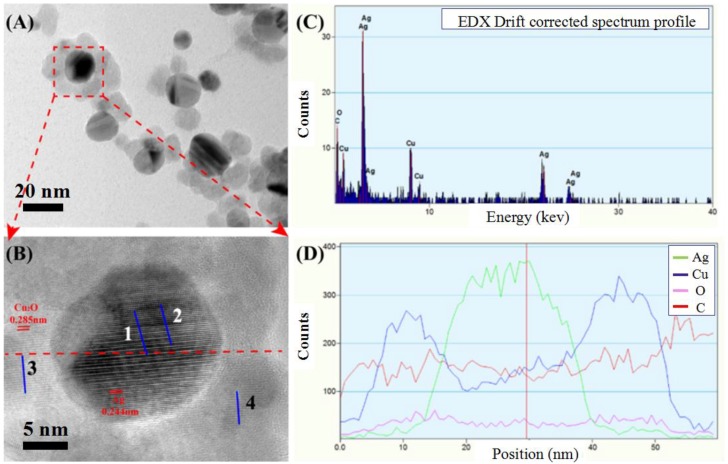
**Figure3.** TEM image (**A**) and high-resolution TEM image (**B**) of the Ag@Cu_2_O-rGO (S-8 mM). The EDS line on the red segment in (**B**) corresponds to the different elements (**C**) and the corresponding element count-position curve (**D**).

**Figure 4:**
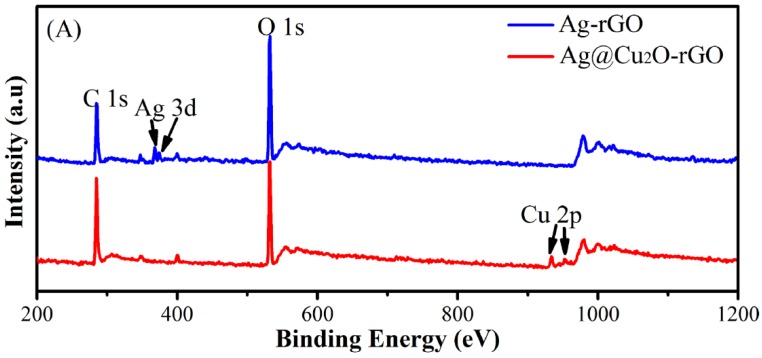

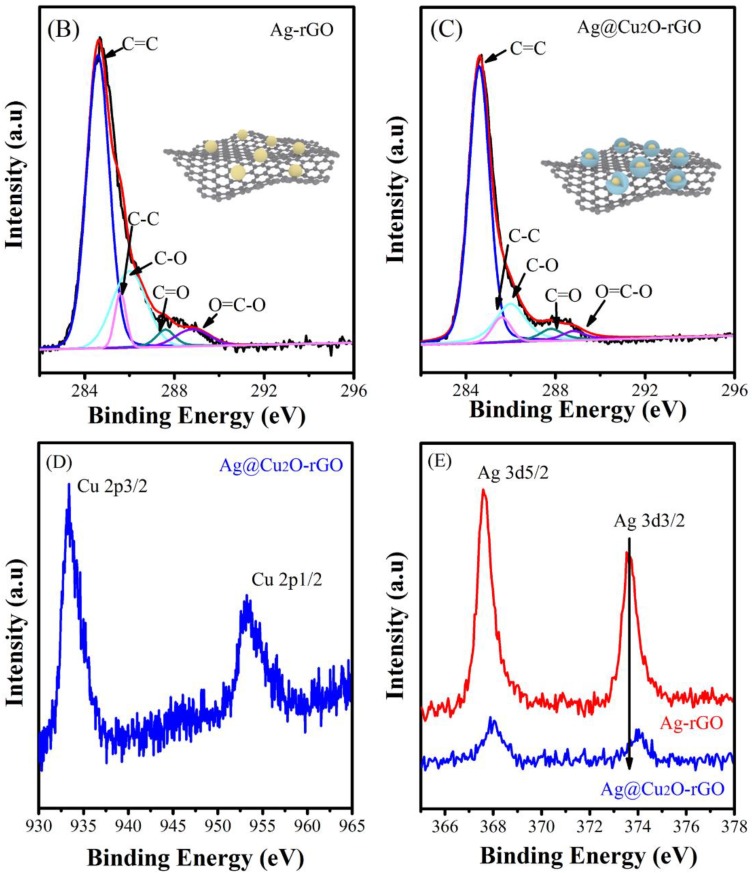
The X-ray photoelectron spectroscopy (XPS) survey spectra (**A**) of Ag-rGO and Ag@Cu_2_O-rGO; high-resolution XPS spectra of C 1s for (**B**) Ag-rGO and (**C**) Ag@Cu_2_O-rGO. The XPS survey spectra of Cu element and Ag element (**D**) in Ag@Cu_2_O-rGO.

High resolution transmission electron microscopy (HRTEM) measurement in Figure 3A,B is used for Ag@Cu_2_O-rGO prepared from Cu(NO_3_)_2_ concentration 8 mM (S-8 mM). The interplanar spacing of the core is 0.244 nm corresponding to the (111) plane of cubic Ag, and the interplanar spacing of shell is about 0.285 nm corresponding to the (200) plane of cubic Cu_2_O, as in Figure S4, X-ray diffraction patterns can further explain the types of elements contained in the structure, as in Figure S5. The energy dispersive spectrometer (EDS) profile (Figure 3C) shows four elements of C, O, Ag, and Cu, and the corresponding line profile (Figure 3D) shows the Ag line profile (green line) is obviously smaller than the line profile of Cu (blue line), indicating that the Cu_2_O shell has been modified and encapsulated by the Ag nanoparticles. The EDS mapping of the Ag@Cu_2_O-rGO nanocomposite is shown in Figure S6. It was observed that the C content in the sample area is significantly higher than in the surrounding area, thus indicating that the Ag@Cu_2_O core-shell structure is grown on rGO. The typical XPS survey scan spectra in Figure 4A confirm the Cu presence in addition to C, Ag, and O elements when Ag-rGO is decorated by Cu_2_O. The intensity of the Ag peaks in Ag@Cu_2_O-rGO decrease in comparison with that of Ag-rGO, which confirms further that the Ag nanoparticles are covered by Cu_2_O [29]. XPS spectra of C 1s for Ag-rGO and Ag@Cu_2_O-rGO in Figure 4B,C are deconvoluted into five carbon peaks centered at 284.6, 285.6, 286.0, 287.6, and 288.8 eV, which are assigned to sp^2^-hybridized carbon (C=C), sp^3^-hybridized carbon (C–C), hydroxyl carbon (C–O), carbonyl carbon (C=O), and carboxyl carbon (O=C–O), respectively [27]. Compared with Ag-rGO, the intensities of the C–O and C–C peaks decrease for Ag@Cu_2_O-rGO. We speculate that the hydroxyl in graphene is replaced by the newly synthesized Cu_2_O nanoparticles and the conjugated graphene networks are re-established. The characteristic peaks of Cu 2p3/2 and Cu 2p1/2 at 932.8 eV and 952.8 eV position are assigned to Cu^+^ and without other peaks are observed in the Figure 4D [30]. This result indicates that all Cu elements in the Ag@Cu_2_O-rGO exist in the form of Cu_2_O. In Figure 4E, the peaks of Ag3d at 367.6 (Ag 3d5/2) and 373.5 eV (Ag 3d3/2) in Ag@Cu_2_O-rGO move to a higher binding energies position than that of Ag3d in Ag-rGO, which is caused by the transfer of free electrons on the Ag surface [22]. For the Ag@Cu_2_O-rGO platform-shell-core structure, rGO with a better conductivity property acts as an electron transfer platform so that it quickly transfers the free electrons on the Ag surface to the Cu_2_O shell or adjacent core-shell structure.

**Figure 5:**
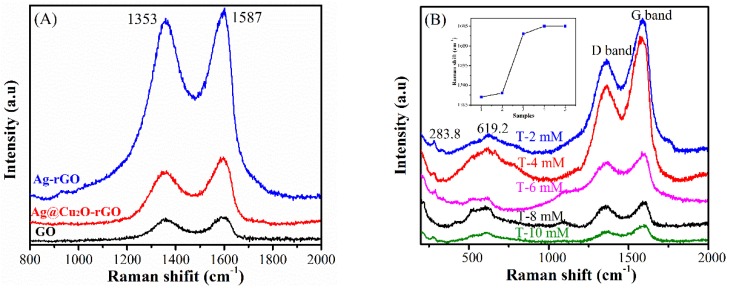
(**A**) Raman spectra of GO, Ag-rGO, and Ag@Cu_2_O-rGO; (**B**) Raman spectra of Ag@Cu_2_O-rGO nanocomposites for S-2 mM to S-10 mM, the inset shows the dependence of the intensity of Ag@Cu_2_O-rGO on Cu_2_O content.

Figure 5A shows the Raman spectra of GO, Ag-rGO, and Ag@Cu_2_O-rGO. The Raman spectra showed two characteristic bands centered at 1353 cm^−1^ (D band) and 1587 cm^−1^ (G band) for graphitic domains. The D band is the defect band, which is based on the presence of sp^3^ hybridized carbon (C–C) atoms. The stretching vibration of the sp^2^ hybridized C=C bond is the main reason for the G band. The ratio of the Raman intensity (*I*_D_/*I*_G_) is usually calculated for the disorder degree of the GO [31]. According to the spectra in Figure 5A, the *I*_D_/*I*_G_ values of the GO and Ag-rGO and Ag@Cu_2_O-rGO are calculated to be 0.93, 0.95, and 0.79, respectively. Compared with GO, the *I*_D_/*I*_G_ increases slightly when Ag is decorated on rGO (that is Ag-rGO) while *I*_D_/*I*_G_ decreases significantly in Ag@Cu_2_O-rGO. It is believed that the increase of *I*_D_/*I*_G_ is caused by the increase of defect when GO is reduced to rGO by sodium citrate in the Ag-rGO formation process [32]. However, when Cu_2_O is introduced to the Ag-rGO system to form the Ag@Cu_2_O-rGO structure, Cu_2_O NPs on the surface of the rGO platform occupied the defect position of rGO and restored the conjugation of rGO platform, which contributes to the significant decrease of the *I*_D_/*I*_G_ intensity ratio. This result also confirms the speculation in XPS analysis. The Fourier transform infrared (FT-IR) spectra also can indicate that most of the oxygen-containing groups in GO have been removed by two reduction reactions in the complex, as in Figure S7. Figure 5B shows the Raman spectra of Ag@Cu_2_O-rGO platform-core-shell structure for different samples (S-2mM to S-10mM). The peaks at 283 and 619 cm^−1^ are assigned to Cu_2_O and the relative intensity increases when the Cu_2_O thickness increases. The G band of rGO shifts to a higher frequency with the increase of Cu_2_O thickness and the peaks center at 1587, 1588, 1603, 1605, and 1605 cm^−1^ for Ag@Cu_2_O-rGO samples S-2mM to S-10mM, as shown in the inset of Figure 5B. This result confirms the charge is transferred from rGO to Cu_2_O. It is known that the rGO cannot generate free electrons by itself but can transfer electrons [33]. Combining with the XPS result of Ag, the peak shift of rGO is caused by the charge transfer between the Ag core and the Cu_2_O shell. In the Ag@Cu_2_O-rGO, rGO acts as an electron transfer platform to make the free electrons on the Ag surface via rGO to the Cu_2_O shell accompanied by peaks shift of G band.

### 3.2. Optical Properties of the Ag@Cu_2_O-rGO Nanocomposites

**Figure 6:**
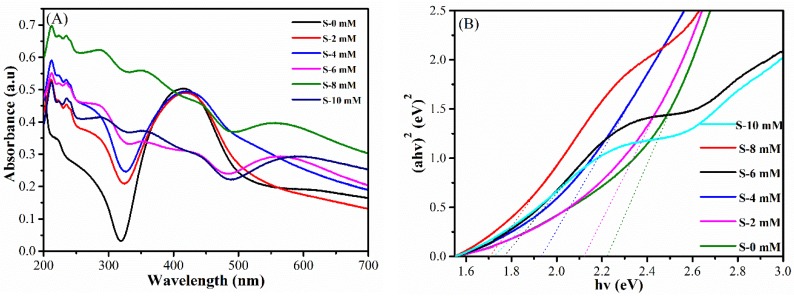
(**A**) UV-VIS absorption spectra of Ag-rGO and the Ag@Cu_2_O-rGO nanocomposites S-2 mM, S-4 mM, S-6 mM, S-8 mM, and S-10 mM; (**B**) the plots of (αhν)^2^ vs. photon energy (hν).

The spectral absorption of nanomaterials reflects the light response of the material directly, and the optical properties of the Ag@Cu_2_O-rGO are characterized by UV-VIS absorption spectroscopy. Figure 6A shows UV-VIS absorption spectroscopy of Ag@Cu_2_O-rGO for samples S-0 mM to S-10mM. Without Cu_2_O shell, the Ag-rGO samples display two significant bands, the band in the range of 250–320 nm arising from the π → π* transition of the C=C bands and the n → π* transition of the C=O bonds in rGO, and the band in the range of 320–500 nm arising from the surface plasmon resonance (SPR) of Ag NPs [34]. When the amount of Cu_2_O is increased, the absorption peak of Ag@Cu_2_O core-shell structure appears gradually at 500–650 nm and the absorption edge shows an obvious red shift with the thickness of the Cu_2_O increases. The SPR band of the silver nanoparticles also shows a significant red shift due to the interfacial effect between the Cu_2_O nanoparticles and the Ag nanoparticles. The band gap of the sample can be roughly calculated based on the relationship between the incident light energy and the absorption coefficient in the band theory [35]. The bandgaps for the Ag@Cu_2_O-rGO samples S-0 mM to S-4 mM are approximately 2.22, 2.12, and 1.90 eV, when the core-shell structure is formed (S-6 mM), the composite structure shows longer wavelength absorbance and the band energy is significantly reduced, as displayed in Figure 6B. The bandgap of the Ag@Cu_2_O-rGO gradually decreases as the Cu_2_O thickness increases, and the optimum bandgap value of 1.71 eV (S-6 mM) was determined. The decreasing of bandgap is attributed to the Schottky effect of Cu_2_O and Ag and the multi-interface coupling in Ag@Cu_2_O-rGO composites. However, the abnormal small increasing of bandgap for the Ag@Cu_2_O-rGO (S-10 mM) is caused by the excess content of Cu_2_O that is not affected by the interface effect.

**Figure 7:**
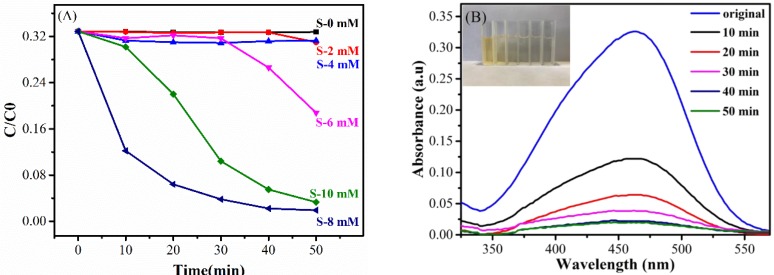
(**A**) Photocatalytic degradation of Methyl Orange (MO) (4 mg/L) under visible-light irradiation at room temperature with different catalysts: Ag-rGO and the Ag@Cu_2_O-rGO nanocomposites S-2 mM, S-4 mM, S-6 mM, S-8 mM, and S-10 mM; (**B**) absorption spectra of the photocatalytic degradation of MO in the presence of Ag@Cu_2_O-rGO (S-8 mM) the inset is the corresponding digital camera photographs of MO dye.

**Figure 8:**
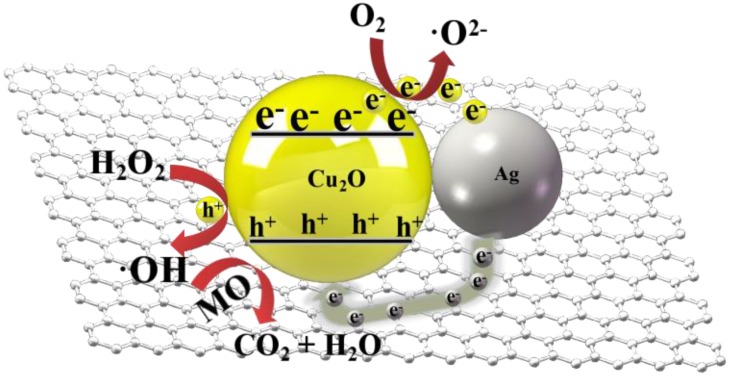
Schematic illustration of the Ag@Cu_2_O-rGO charge transfer in the photocatalytic degradation process of MO.

The photo-substance interaction ability of the Ag@Cu_2_O-rGO nanocomposites are evaluated by examining the photocatalytic degradation of an MO (4 mg/L) aqueous solution with visible-light irradiation, as shown in Figure 7A. The change in the amount of MO in an aqueous solution is monitored by detecting the maximal absorption in the UV-VIS spectra at 463 nm, as shown in Figure 7B. The degradation percentages of Ag-rGO and Ag@Cu_2_O-rGO with different Cu_2_O shell thicknesses are S-8 mM (94.0%) > S-10 mM (90.3%) > S-6 mM (43.0%) > S-4 mM (6.3%) ≈ S-2 mM (6.2%) > S-0 mM (3.0%). Obviously, the photocatalytic activity of Ag@Cu_2_O-rGO dramatically increases as the Cu_2_O thickness increases from 0 mM to 8 mM and the optimum photocatalytic activity is obtained by S-8 mM. Comparison of catalytic performance of Ag@Cu_2_O-rGO and Ag@Cu_2_O structures showed that Ag@Cu_2_O-rGO (S-8 mM)’s catalytic ability is better than Ag@Cu_2_O as shown in Figure S8. Analysis of the catalytic mechanism of Ag@Cu_2_O-rGO nanocomposites is based on photocatalytic theory of semiconductors and metals, and the unique nature of the structure as depicted in Figure 8. In Ag@Cu_2_O core-shell, the semiconductor of Cu_2_O energizes the photogenerated electron-hole pairs under illumination of light with energy greater than 1.71 eV. The electrons transfer from the conduction band of Cu_2_O to the Fermi level of the Ag NPs leaving holes in the valence band of Cu_2_O, since the work function of Ag is 4.26 eV which is lower than Cu_2_O (~5.0 eV) [36]. Based on the unique nature of the Ag@Cu_2_O-rGO nanocomposites, the vibrational frequency of the G band moved to high frequencies when Cu_2_O particles was introduced to the system, which indicates that electrons move from rGO to Cu_2_O. Therefore, the rGO as the charge-transfers platform makes the electrons transfer quickly from Ag NPs to the Cu_2_O shell layer or adjacent core-shell structure to increase photon utilization efficiency. The results above indicate that the Ag@Cu_2_O-rGO platform-core-shell nanocomposite has an excellent light–matter interaction.

## 4. Conclusions

In summary, we designed a heterostructure of Ag@Cu_2_O-reduced graphene oxide (rGO) with the core-shell complex-structure. The rGO nanosheets act as a sheet platform and the Ag@Cu_2_O core-shell nanoparticles are decorating the surface of the rGO, which forms a 2D+0D heterogeneous structure. Cu_2_O nanoparticles occupy the defect positions on the surface of the rGO platform and restore the conjugation of the rGO structure, which contributes to the significant decrease of the *I*_D_/*I*_G_ intensity ratio. The rGO platform can not only bridge the isolated nanoparticles together but also can quickly transfer the free electrons arising from the Ag core to the Cu_2_O shell to improve the utilization efficiency of photogenerated electrons. The broadened absorption of visible light and high efficient photocatalytic activity to MO in Ag@Cu_2_O-rGO indicates the strong light–matter interactions.

## Supplementary Materials

The following are available online at http://www.mdpi.com/2079-4991/8/6/444/s1, Figure S1: Schematic flow chart of the synthetic procedure for Ag@Cu_2_O-rGO nanostructures, Figure S2: SEM image of GO, Figure S3: Particle size distribution (A) Internal Ag NPs diameterof S-6 mM; (B) External Cu_2_O shell thickness of S-6 mM; (C) Internal Ag NPs diameterof S-10 mM;(D) External Cu_2_O shell thickness of S-10 mM, Figure S4: the drawing of crystal surface spacing correspond to HRTEM in Figure 1C, Figure S5: X-Ray diffraction patterns of GO, Ag-rGO and Ag@Cu_2_O-rGO, Figure S6: EDS elemental mapping images (C, Cu and Ag) of corresponding red line square for Ag@Cu_2_O-rGO nanocomposites, Figure S7. FTIR spectra of GO and Ag@Cu_2_O-rGO, Figure S8. Photocatalytic degradation of MO (4 mg/L) under visible-light irradiation at room temperature with different catalysts: Ag@Cu_2_O and the Ag@Cu_2_O-rGO(S-8 mM) nanocomposites.
