# Supplementary material for: In Situ Synthesis of Ag@Cu2O-rGO Architecture for Strong Light-Matter Interactions
Source: Nanomaterials (Basel). 2018 Jun 17;8(6):444. doi: 10.3390/nano8060444 (PMC6027245; doi:10.3390/nano8060444)
Supplement: Supplementary file 1 [file nanomaterials-08-00444-s001.pdf]

In Situ synthesis of Ag@Cu2O-rGO architecture for Strong Light-Matter Interactions

Shuang Guo[1, 2], Yaxin Wang[1,*], Fan Zhang[1], Renxian Gao[1], Maomao Liu[1], Lirong Dong[1], Yang Liu[2], Yongjun Zhang[2,*], Lei Chen[3,*]

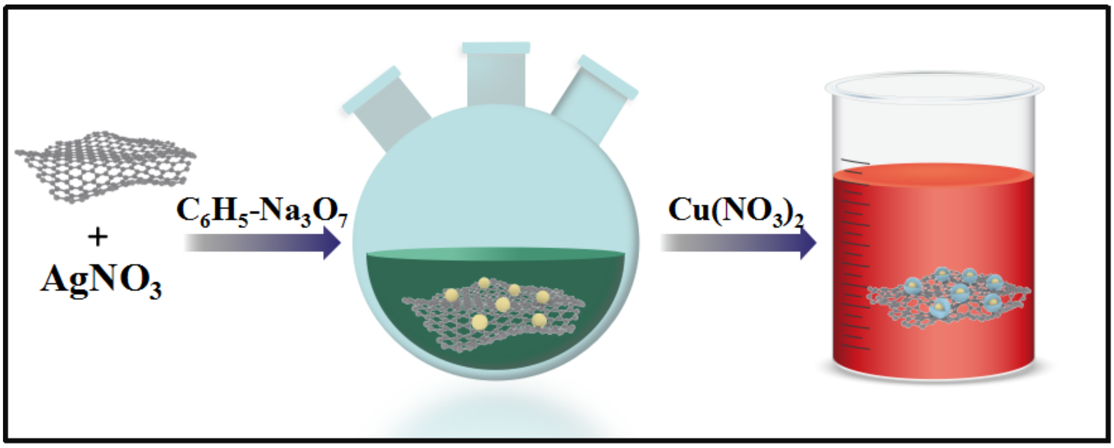

Figure S1. Schematic flow chart of the synthetic procedure for Ag@Cu_2O-rGO nanostructures.

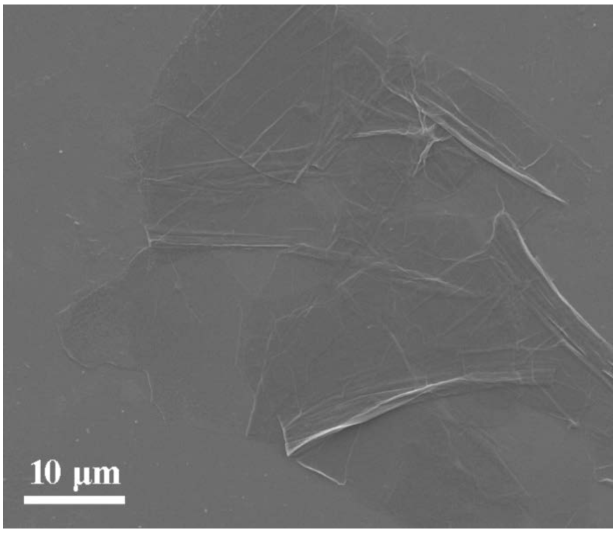

Figure S2. SEM image of GO.

The synthesis of Graphene oxide:

Graphene oxide nanosheets were synthesized by two-step oxidation of graphite powder summarized from Hummer's and Offeman's method[1]. This method is divided into two stages, the preparatory oxidation stage and the deep oxidation stage. At preparatory oxidation stage, firstly, 2.5 mg $K_2S_2O_8$ and 2.5 mg P_2O_5 were put into cone bottle with a geometrical size of 50 mL, and 12.5 mL of H_2SO_4 was also flood into the cone bottle, and

magnetic stirring was performed to dissolve the powder completely. Then 3 mg of graphite powder was dissolved in the cone bottle, and the cone bottle was placed in the thermostat water bath with mechanical agitation and a condenser at 80 °C for 5 h. After that, the solution of cone bottle completely poured into 1000 mL beaker, and the beaker was filled with deionized water, and the solution of beaker was filtered into four filter cakes. Finally, the sample obtained in the above procedure was placed in a petrie dish and was dried at room temperature for 24 h. At the deep oxidation stage, in a typical synthesis, 40 mL of H_3PO_4 was put into 500 mL cone bottle, and 360 mL concentrated sulfuric acid was slowly injected into the cone bottle with a glass rod drain, and the as-prepared obtained in the previous process was added to the cone bottle by magnetic stirring for 1 h. Then, the cone bottle was placed in the thermostat water bath (<5 °C) and 18 g of $KMnO_4$ was slowly added with stirring, and the cone bottle was heated for 12 h at 50 °C by a thermostat water bath. The as-prepared sample solution was stirred for 5 days after the solution was cooled to room temperature. Next, the solution in the cone bottle was poured into 2000 mL beaker with 400 mL ice-water mixture, and 12 mL H_2O_2 (30 wt%) was slowly added into the beaker, and the solution was magnetic stirred for 2 h at room temperature. Finally, the solution was centrifuged 15 min at 9500 rpm, and the as-prepared sample was washed by H_2SO_4, HCl, and deionized water until the pH of the sample is neutral, and the sample was freeze dried 5 days. 0.4 g graphene oxide nanosheep were formed by this two stages.

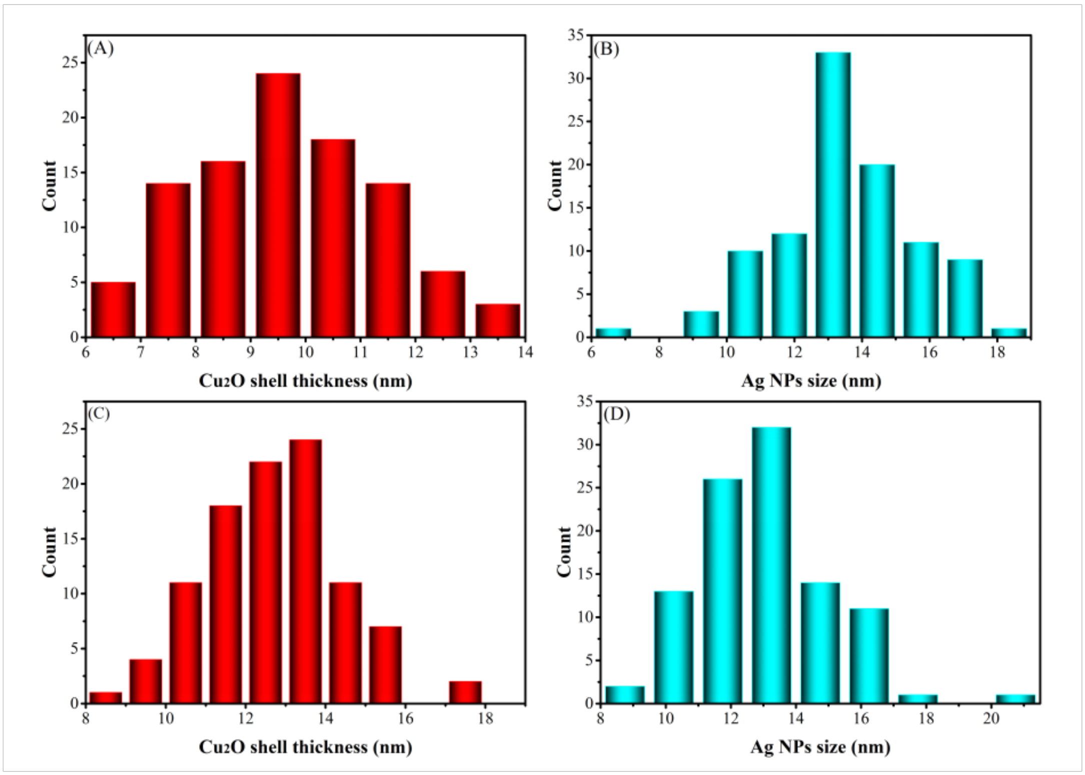

Figure S3. Particle size distribution (A) Internal Ag NPs diameterof S-6 mM; (B) External Cu2O shell thickness of S-6 mM; (C) Internal Ag NPs diameterof S-10 mM;(D) External Cu2O shell thickness of S-10 mM.

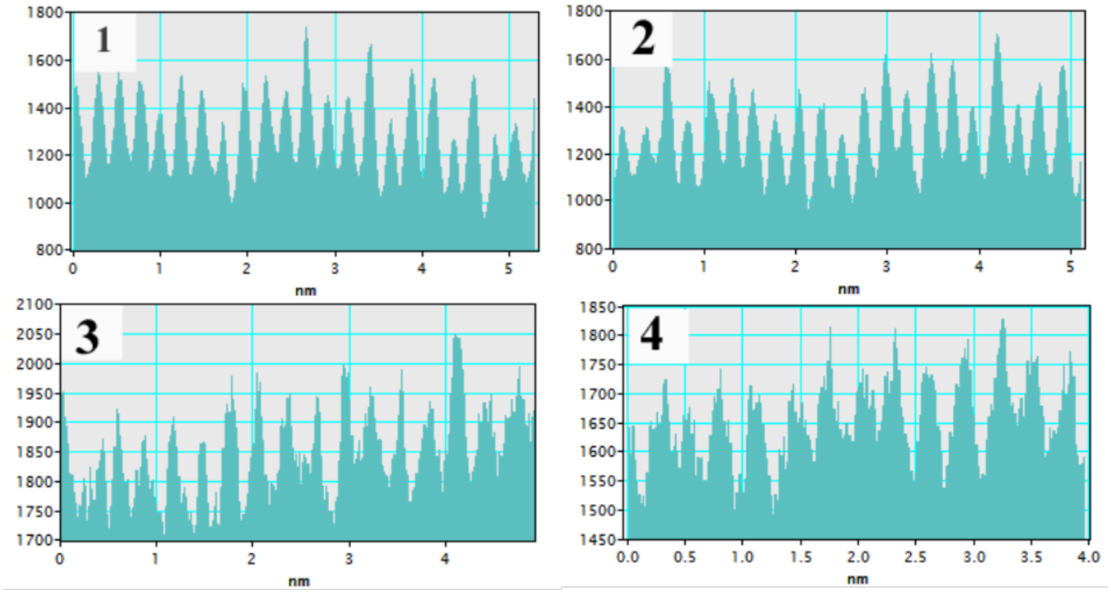

Figure S4. the drawing of crystal surface spacing correspond to HRTEM in Figure 1C.

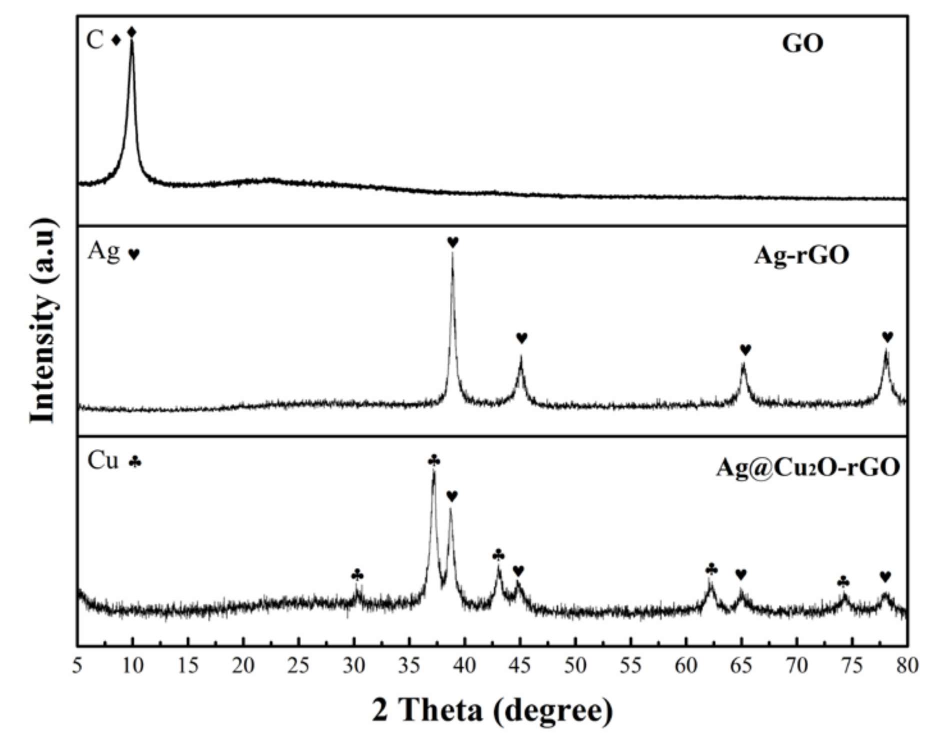

Figure S5. X-Ray diffraction patterns of GO, Ag-rGO and Ag@Cu_2O-rGO.

Figure S4 shows the X-ray diffraction (XRD) patterns of GO, Ag-rGO and Ag@Cu_2O-rGO. For GO, a sharp peak near 9.9°is observed, which corresponds to the characteristic peak of the (001) plane. In the structure of Ag-rGO， all the diffraction peaks are readily indexed to Ag (JCPDS 65-2871). The peaks of 38.9°, 45.1°, 65.2°, and 78.1° correspond to (111), (200), (220) and (311) faces of the Ag, respectively. Moreover, and no typical diffraction peak of graphene (001) is observed, accompanied with the disappearance of the peak at 9.9° for the GO. The main reason for this phenomenon may be that the content of rGO in the structure is too small

[2]. In the pattern of Cu_2O for the structure of Ag@Cu_2O-rGO, all the peaks can be indexed to cubic Cu_2O for the patterns of JCPDS 65-3288.

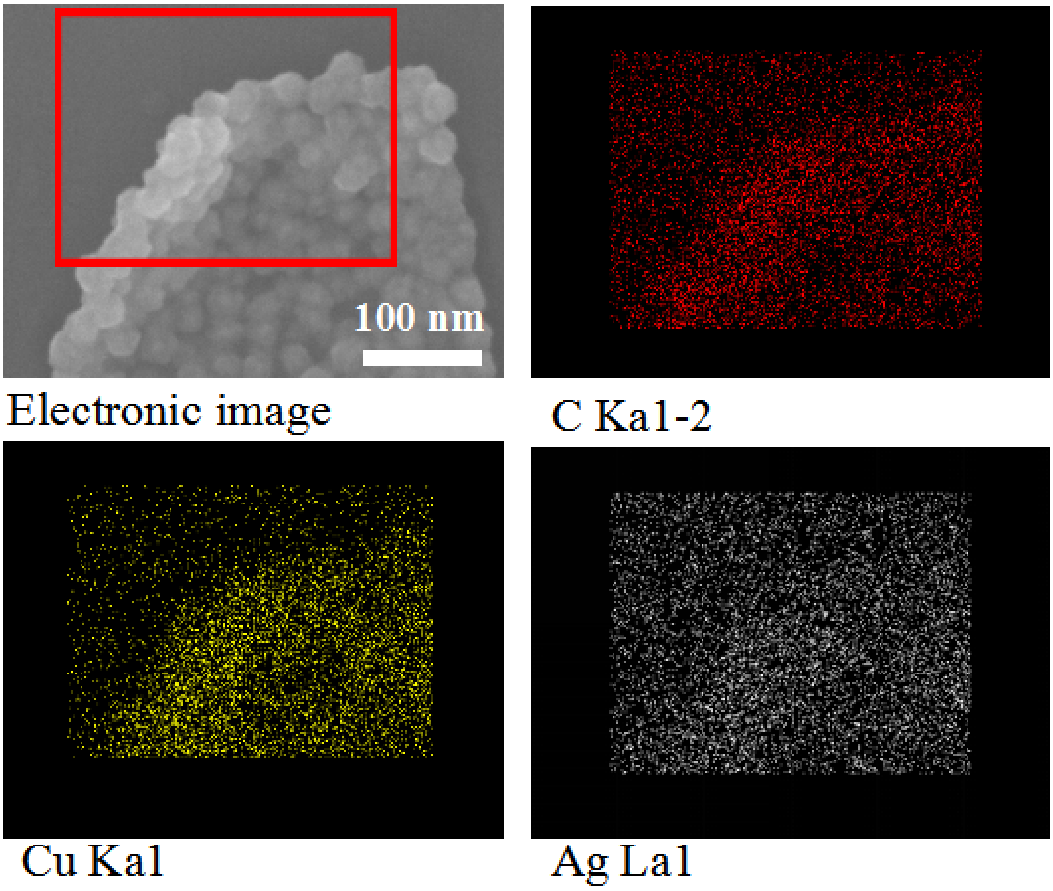

Figure S6. EDS elemental mapping images (C, Cu and Ag) of corresponding red line square for Ag@Cu_2O-rGO nanocomposites.

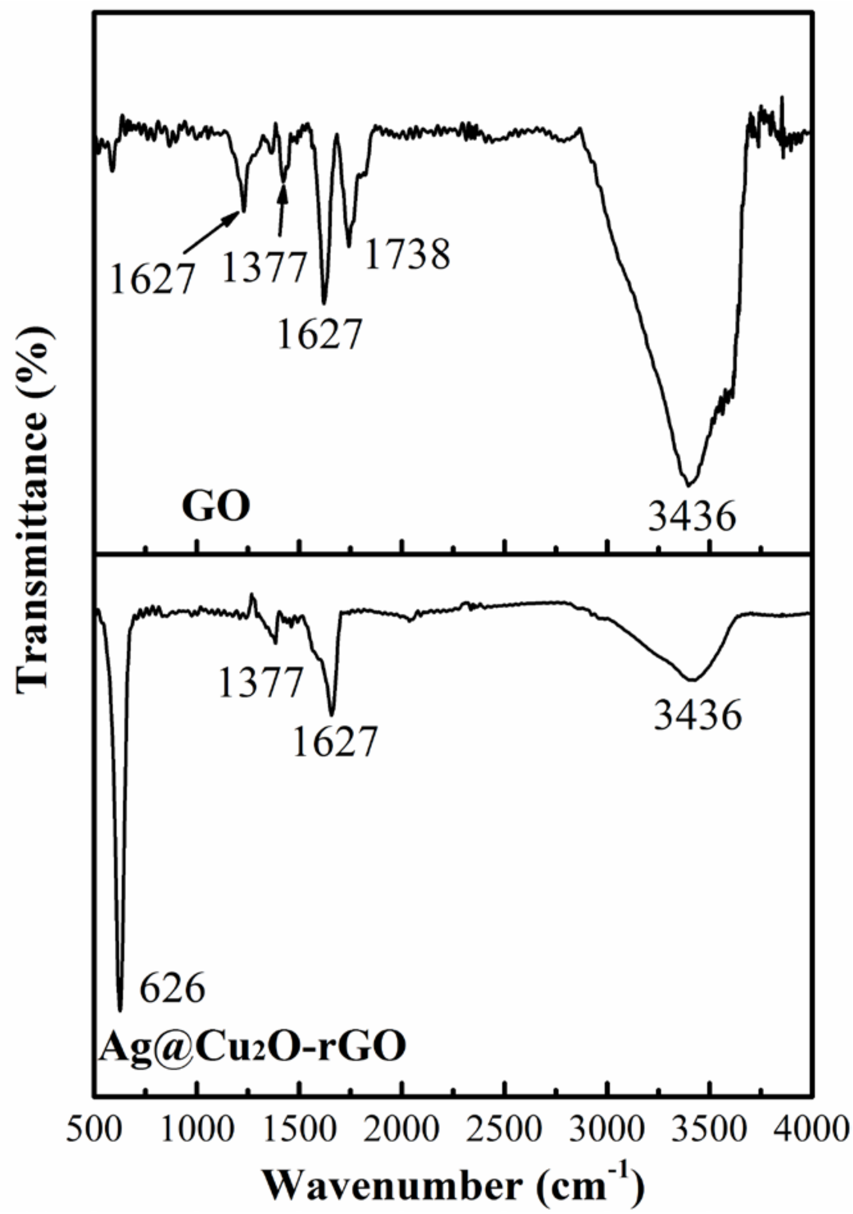

Figure S7. FTIR spectra of GO and Ag@Cu_2O-rGO.

Figure S6 shows the Fourier-transform infrared (FTIR) spectra of the synthesized GO and Ag@Cu_2O-rGO. Obviously, GO shows abundant oxygen-containing groups. The stretching vibration of the O-H group, the carbonyl stretching of C=O and the skeletal vibrations of the graphite domains are located at 3436, 1738, and 1627 cm^{-1}, respectively[2]. In the case of Ag@Cu_2O-rGO, the strength of oxygen-containing functional groups is significantly reduced. It is noteworthy that there is a new vibrational peak at 626 cm^{-1}, which is mainly attributed to the Cu-O stretching vibration in the Cu_2O phase. The phenomena above indicate that most of the oxygen-containing groups in GO have been removed by two reduction reactions and the presence of Cu_2O is also confirmed in the complex.

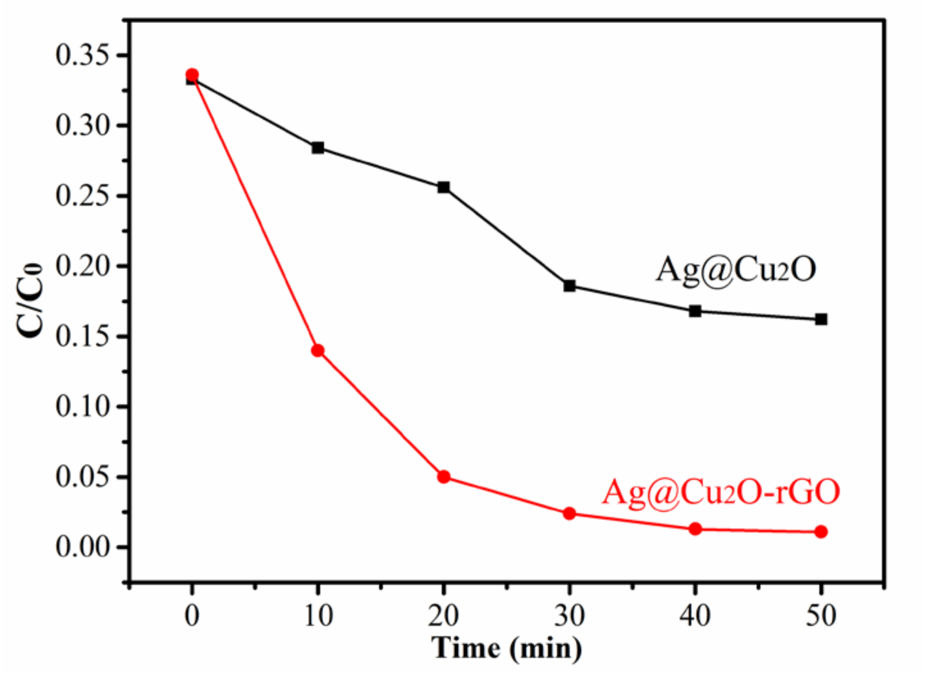

Figure S8. Photocatalytic degradation of MO (4 mg/L) under visible-light irradiation at room temperature with different catalysts: Ag@Cu_2O and the Ag@Cu_2O-rGO(S-8 mM) nanocomposites.

The catalytic performance of Ag@Cu_2O-rGO (S-8 mM) and Ag@Cu_2O was tested under the same conditions as shown in the Figure. S7. The Preparation of Ag@Cu_2O nanoparticles with a similar thickness of Cu_2O was based on a previously reported synthesis method [3]. The comparison result shows that Ag@Cu_2O-rGO (S-8 mM)'s catalytic ability is better than Ag@Cu_2O, this result also claimed that the platform of rGO in the composite structure plays an important role in the catalytic process.

References

1. Marcano, D.C.; Kosynkin, D.V.; Berlin, J.M.; Sinitskii, A.; Sun, Z.; Slesarev, A.; Alemany, L.B.; Lu, W.; Tour, J.M. Improved Synthesis of Graphene Oxide. ACS Nano **2010**, *4*, 4806–4820.

2. Xu, L.; Zhang, F.; Song, X.; Yin, Z.; Bu, Y. Construction of reduced graphene oxide-supported Ag-Cu2O composites with hierarchical structures for enhanced photocatalytic activities and recyclability. J. Mater. Chem. A **2015**, *3*, 5923–5933.

3. Chen, L.; Liu, M. M.; Zhao, Y.; Kou, Q. W.; Wang, Y. X.; Liu, Y.; Zhang, Y. J.; Yang, J. H.; Young, M. J. Enhanced Catalyst Activity by Decorating of Au on Ag@Cu_2O Nanoshell. Applied Surface Science 2018, 435,72–78.
